# Advancing Nurse Practitioner Integration in Health Systems: Contextualizing Porat-Dahlerbruch’s Taxonomy for Global Adaptation: Comment on "Development of a Taxonomy of Policy Interventions for Integrating Nurse Practitioners Into Health Systems"

**DOI:** 10.34172/ijhpm.9398

**Published:** 2025-12-03

**Authors:** Doris Grinspun

**Affiliations:** Registered Nurses’ Association of Ontario (RNAO), Toronto, ON, Canada.

**Keywords:** Nurse Practitioners, Health Policy, Workforce Integration, Implementation Science, Primary Healthcare, Interprofessional Team

## Abstract

This commentary examines the contribution of Porat-Dahlerbruch and colleagues’ taxonomy of policy interventions for integrating nurse practitioners (NPs) into health systems. Developed through stakeholder interviews in Israel, the taxonomy proposes a structured, multi-level framework—macro, meso, and micro—for guiding NP integration. It bridges the gap between theory and practice, providing policy-makers, educators, administrators, and researchers with a practical, evidence-informed tool for reform. The commentary highlights the taxonomy’s alignment with global implementation frameworks and identifies opportunities for further development, including cross-national validation, end-user engagement, robust evaluation metrics, digital health integration, and explicit equity strategies. By embracing these opportunities, the taxonomy can evolve into a global resource for strengthening NP roles and advancing interprofessional collaboration. As NPs become increasingly essential to primary and advanced care, especially in underserved settings, strategic integration is imperative for building resilient and equitable health systems.

## Introduction

 The growing demand for accessible, high-quality, and cost-effective healthcare has placed the health workforce at the centre of policy reform in nearly every country. Among the most promising contributors to sustainable health system strengthening are nurse practitioners (NPs), whose advanced clinical education and collaborative practice capabilities position them to deliver primary, community and specialised care. Across the globe, evidence of NP effectiveness continues to accumulate, yet the processes for integrating NPs into health systems vary widely, presenting timely opportunities for shared learning and innovation.

 In this context, the recent article by Porat-Dahlerbruch et al, “*Development of a Taxonomy of Policy Interventions for Integrating Nurse Practitioners into Health Systems*,”^1^ represents a strong advancement. The authors present a comprehensive taxonomy of 19 policy interventions, drawn from 25 in-depth interviews with four professional groups—policy-makers, organisational leaders, physicians, and NPs—in the Israeli healthcare system. Grouped by system level (macro, meso, micro), this structured framework proposes targeted, multilevel action.

 This commentary recognizes the significance of this contribution, highlights how it aligns with broader policy and implementation frameworks, and invites further exploration and adaptation across diverse health system contexts worldwide.

## Advancing Policy Design Through Structure

 A key contribution of Porat-Dahlerbruch and colleagues’ taxonomy is its effort to bridge the gap between theory and practice in NP workforce integration. While the existing literature documents barriers (eg, role ambiguity, physician resistance, regulatory constraints) and enablers (eg, team support, mentorship, clear governance), few translate this knowledge into a coherent, actionable policy framework. This taxonomy provides a descriptive map and a practical toolkit for change.

 Each level of the taxonomy corresponds to a distinct sphere of influence, creating opportunities for targeted interventions that can be pursued independently or in concert:

- Macro-level interventions—such as licensure, professional marketing, education pipelines, funding models, and national collaboration—establish the legal, economic, and societal conditions that legitimise and mobilise NPs.
- Meso-level strategies—including internal policies, organisational readiness, NP leadership development, and institutional messaging—shape the structural integration of NPs within hospitals, clinics, and community health organisations.
- Micro-level policies—covering team-based dynamics such as communication norms, mentorship, and role socialisation at the unit level—address the day-to-day practice culture, an essential yet often underexplored element in successful integration.

By capturing the interdependencies across these levels, the taxonomy offers a nested model that mirrors the realities of healthcare delivery. It invites policy-makers, educators and organisational leaders to design reforms that can be phased in or implemented in parallel, enabling both incremental progress and system-wide transformation.

## Opportunities for Further Exploration

 Porat-Dahlerbruch and colleagues’ taxonomy is both practical and conceptually strong, providing a valuable foundation for advancing NP integration policy. Equally important, it opens the door to further work that could broaden its relevance and deepen its impact across diverse health system contexts. Building on its strengths, several areas emerge as promising opportunities for exploration:

###  1. International Context and Comparability

 In many countries, NPs are emerging both as a solution to primary care physician shortages and a contribution to holistic patient care, cost-effective management, and team-based care. For those seeking to apply Porat-Dahlerbruch and colleagues’ taxonomy in their own health system, it is important to recognize that the framework reflects the Israeli context, where the NP role is relatively new and shaped by a centralized public health system. In high-income countries (HICs) with more established NP workforces, adaptation may be needed to reflect mature regulatory and educational infrastructures, decentralized governance and a larger role for the private sector.

 In low-income countries (LICs) and low- and middle-income countries (LMICs), where NPs are often introduced to address primary care gaps, additional challenges may arise, such as limited infrastructure, constrained faculty capacity, or weaker institutional support. These differences suggest that while the taxonomy offers a valuable foundation, its application may benefit from adaptation to reflect local system characteristics. The work of Scanlon et al makes an important contribution to the understanding of how these advanced nursing roles are operationalized in relation to education, practice, and regulation in LMIC. They find that while the main reason for developing such roles in LIC and LMIC was to care for underserved populations, in many HIC the top reason was to address physician or specialty care shortages.^2^

###  2. Implementation and Evaluation Metrics

 While the taxonomy offers a strong conceptual framework for policy design, its utility could be enhanced by pairing each intervention with practical measures for tracking progress and impact. Clear benchmarks are important for policy-makers and organisations to monitor implementation, assess outcomes, and make the case for continued investment.

 Future development could involve creating key performance indicators linked to each policy domain—for example, NP retention rates, time to integration, team satisfaction scores, patient access measures, and quality-of-care outcomes. Such metrics would enable systematic evaluation, support evidence-informed adjustments, and demonstrate the value of NP integration over time.

 Htay and Whitehead’s 2021 systematic review^3^ is an illustrative example: their comparative analysis of 13 randomised controlled trials conducted across HIC examined NP-led care in primary, secondary, and specialist care settings involving both adult and paediatric populations. Outcomes assessed included patient satisfaction, waiting times, chronic disease control, and cost-effectiveness—particularly when compared directly with physician-led or usual care. Insights from such rigorous evaluations could guide the development of robust indicators embedded within Porat-Dahlerbruch and colleagues’ taxonomy.

 Equally important is the inclusion of patient, caregiver, and community perspectives as part of evaluation. Their input is central to understanding legitimacy, relevance, and the real-world impact of NP-led care. Engaging patients and the public can illuminate dimensions such as access, trust, continuity, and satisfaction—elements not fully captured by professional or institutional metrics. End-user involvement has historically been limited in health policy design, and this taxonomy provides an opportunity to address that gap. Approaches such as participatory co-design, focus groups, and surveys can ensure the framework reflects public priorities and strengthens its legitimacy in implementation.

 Bird et al^4^ have shown that embedding end-user engagement throughout design, delivery, and evaluation enhances the relevance and uptake of innovations. Similarly, Thompson et al^5^ highlight how NPs themselves face gaps in evaluating their specialty practice compared to medicine, underscoring the need for consistent, patient-centred evaluation strategies. Embedding such perspectives directly into implementation and evaluation metrics ensures that reforms are not only technically sound but also socially legitimate and sustainable.

###  3. Integration of Digital and Virtual Care Models

 As healthcare delivery increasingly incorporates digital tools, there is an opportunity to expand the taxonomy to reflect the realities of contemporary NP practice. Telehealth, remote patient monitoring, electronic health records, and artificial intelligence-enabled decision support are now integral to many clinical environments, particularly in rural, remote, and primary care settings where these tools can bridge access gaps. Including explicit consideration of digital readiness within the taxonomy could further enhance its alignment with evolving models of care.

 Future development could incorporate policy supports for virtual NP practice, such as reimbursement mechanisms, training standards, and equitable access to digital infrastructure. This aligns with the American Association of Nurse Practitioners’ identification of virtual care as one of its five critical healthcare trends to watch in 2025.^6^ Emerging evidence, including scoping reviews of NP-led telehealth, highlights both patient acceptability and improved access to services. Embedding these elements into the taxonomy would position it as a forward-looking framework capable of guiding NP integration in a technology-enabled healthcare landscape.

###  4. Health Equity Considerations

 While the taxonomy indirectly supports improved access through NP integration, its future iterations could explicitly embed equity as a guiding principle across all levels. Doing so would help ensure that policy interventions address the persistent disparities faced by underserved populations—whether defined by geography, socio-economic status, ethnicity, sex and gender diversity, or health condition. In Israel, this includes Arab Palestinian and Jewish ultra-Orthodox (Haredi) communities and persons with severe mental illness^7^; internationally, similar inequities affect Indigenous, racialised, rural, and economically disadvantaged populations.

 Future work could incorporate targeted equity strategies into deployment planning, education outreach, and financial supports, so that reforms actively contribute to narrowing gaps in access and outcomes. The United Nations Sustainable Development Goals provide a strong global framework for anchoring this equity focus, and existing models—such as the Registered Nurses’ Association of Ontario’s NP Task Force recommendations^8^— demonstrate how NP policy can be intentionally aligned with social justice objectives. By embedding equity considerations directly into the taxonomy, its relevance and impact could be maximised for diverse health systems worldwide.

## Situating Porat-Dahlerbruch et al Within Global Taxonomies and Frameworks

 This work is strengthened by its alignment with, and contribution to several implementation and health system frameworks:

- **EPOC Taxonomy (Cochrane)** – Classifies health system interventions broadly across governance, financial, and delivery domains, but lacks detail specific to professional roles.^9^
- **ERIC Taxonomy** – Contains 73 discrete implementation strategies, many of which (eg, “develop academic partnerships,” “revise professional roles”) are reflected in Porat-Dahlerbruch and colleagues’ taxonomy.^10^
- **World Health Organization (WHO) Building Blocks Framework** – Maps system functions such as workforce and service delivery, and affords important insights for linking research to action,^11,12^ but does not explicitly address role integration strategies.^12^
- **Supporting the Use of Research Evidence (SURE) Framework** – Used effectively in LMIC as a diagnostic tool for identifying implementation barriers.^13^ Although its current use is more limited, it could complement the taxonomy by highlighting areas for refinement and avoiding shortcomings.
- **Theoretical Domains Framework (TDF)** – Focuses on behaviour change in implementation science and can be used to operationalise team-level interventions identified in the taxonomy’s micro-level category.^14^

Porat-Dahlerbruch and colleagues’ taxonomy serves as a mid-range, role-specific framework that connects real-world stakeholder experiences with systems thinking and implementation theory. It addresses a gap in the current ecosystem by granting both conceptual clarity and practical applicability to NP integration. As the authors highlight in a recent publication, it provides “an inventory to aid in designing policies to better integrate nurse practitioners into health systems,” correctly recognising that “the integration of the nurse practitioner workforce could be conceptualized as an implementation issue.”^15^

## Conclusion

 Porat-Dahlerbruch et al have provided the global health community with a practical and policy-relevant taxonomy for NP integration. Its clarity, strong grounding in stakeholder perspectives, and multi-level structure make it both a conceptual advancement and a pragmatic tool for decision-makers. The taxonomy’s emphasis on structured, multi-level action aligns closely with contemporary health system needs and offers an evidence-informed foundation for designing reforms.

 The framework’s design creates distinct opportunities for different stakeholders:

- **Policy-makers** can use it as a roadmap for NP regulation and integration, particularly in jurisdictions initiating NP licensure, identifying which policy levers are most relevant at each stage of workforce development.
- **Educators** can draw on it to strengthen system-level support for graduate readiness and role clarity, guiding curriculum development and professional learning.
- **Administrators** can apply it as a structured guide for organisational change, enabling targeted interventions at macro, meso, and micro levels.
- **Researchers** can use it to inform longitudinal studies that evaluate the impact of policy interventions on workforce integration, patient outcomes, and system performance.

Its potential may be further realised by integrating end-user perspectives, digital health innovations, robust evaluation metrics, and equity-focused strategies into future adaptations, enabling it to evolve into a global resource for strengthening NP roles and advancing interprofessional collaboration. As NPs become a linchpin of primary and advanced care models, especially in underserved and resource-stretched settings, strategic integration is essential, and with continued refinement, international adaptation, and rigorous evaluation, the taxonomy has the potential to meaningfully aid in shaping large-scale NP-related reforms.

 Maximising this potential will require embedding end-user involvement across all phases of NP role advancement—from role development and enhancement to integration into workflows and health systems, as well as ongoing monitoring, research, and evaluation. Although end-user co-design remains limited in the literature, it represents an important pathway toward achieving our shared goal of effective, sustainable NP integration in health systems worldwide.

## Disclosure of artificial intelligence (AI) use

 Not applicable.

## Ethical issues

 Not applicable.

## Conflicts of interest

 The author is the Chief Executive Officer of Registered Nurses’ Association of Ontario (RNAO). RNAO receives funding from the Ontario Ministry of Health.
